# Lizards as Silent Hosts of *Trypanosoma cruzi*

**DOI:** 10.3201/eid2806.220079

**Published:** 2022-06

**Authors:** Carezza Botto-Mahan, Juana P. Correa, Raúl Araya-Donoso, Francisca Farías, Esteban San Juan, Nicol Quiroga, Ricardo Campos-Soto, Claudio Reyes-Olivares, Daniel González-Acuña

**Affiliations:** Universidad de Chile, Santiago, Chile (C. Botto-Mahan, F. Farías, E. San Juan, N. Quiroga);; Universidad San Sebastián, Concepción, Chile (J.P. Correa);; Arizona State University, Tempe, Arizona, USA (R. Araya-Donoso);; Universidad Viña del Mar, Viña del Mar, Chile (R. Campos-Soto);; Universidad Andrés Bello, Santiago (C. Reyes-Olivares);; Universidad de Concepción Campus Chillán, Chillán, Chile (D. González-Acuña)

**Keywords:** Chagas disease, lizards, silent hosts, Trypanosoma cruzi, American trypanosomiasis, vector-borne infections, transmission, reptiles, neglected diseases, zoonoses, Chile

## Abstract

We assessed 4 lizard species in Chile for *Trypanosoma cruzi*, the causative agent of Chagas disease, and 1 species for its ability to transmit the protozoan to uninfected kissing bugs. All lizard species were infected, and the tested species was capable of transmitting the protozoan, highlighting their role as *T. cruzi* reservoirs.

Chagas disease is one of the most neglected vectorborne diseases, infecting 6–7 million persons worldwide; 70 million persons are at risk for infection (*1*), and the disease is a concern in several nonendemic countries (*2*). The etiologic agent is *Trypanosoma cruzi*, a zoonotic protozoan maintained in the Americas by wild and domestic mammals and transmitted by hematophagous triatomine vectors (kissing bugs) (*3*,*4*). Infection of mammals occurs by contamination of broken skin or mucous membranes with the protozoan in kissing bug feces, by congenital transmission, and orally when feeding on infected kissing bugs (or their feces) or other infected mammals (*3*,*4*). Kissing bugs become infected mainly when feeding on infected mammals (*3*).

More than 150 species of wild mammals in the Americas are naturally infected with *T. cruzi* protozoa; some of these hosts are relevant in the maintenance and interplay of the domestic and wild transmission cycles of Chagas disease (*3*,*4*). Although the role of mammals in *T. cruzi* transmission has been studied, less is known about the relevance of nonmammalian vertebrates (*5*). Reptiles have been reported as blood meal sources of kissing bugs, but their status as hosts for *T. cruzi* protozoa is not well documented (*6*).

Reptiles have been described as natural hosts of some Trypanosomatid species transmitted by fly species (*7*). Although studies have shown how lizards could become experimentally infected by *T. cruzi* protozoa (*8*,*9*) and one showed an association between kissing bug infection and lizard abundance (*10*), most studies have not included reptiles as potential vertebrates involved in persistence and transmission of *T. cruzi* protozoa. To determine persistence of vectorborne infections in natural systems, it is essential to describe and characterize all host species directly (i.e., naturally infected species) or indirectly (i.e., vector blood meal sources) involved and evaluate their contribution to kissing bug infection.

We examined *T. cruzi* infection in 4 lizard species from the Pacific coast of Chile coexisting with kissing bug species. We also evaluated the competence of the most abundant lizard species to transmit *T. cruzi* protozoa to kissing bugs.

## The Study

We conducted capturing and processing procedures after approval was obtained from the Institutional Committee for the Care and Use of Animals, University of Chile (permit 19275-FCS-UCH), the Agricultural and Livestock Service of Chile (permits 805/2018, 334/2019, and 4944/2019), and the National Forestry Corporation of Chile (permit 66/2018). We captured lizards at 3 sites in interior valleys and 2 Pacific islands in the arid‒semiarid Mediterranean ecosystem of South America, where lizards naturally occupy the same microhabitats as kissing bugs (*Mepraia* spp.).

Depending on the species, we obtained blood from lizards in the field by using tail clipping and releasing (*Microlophus atacamensis*, Pacific Atacama racerunner) or in the laboratory by using tissue/organ extraction (*Liolaemus platei*, Plate’s lizard; *Liolaemus fuscus*, dark lizard; *Garthia gaudichaudii*, Chilean marked gecko). We kept lizards for 1 week in the laboratory in terraria containing food, water, and light. Before processing, Plate’s lizards were subjected to xenodiangoses with 3 axenic second nymph stage kissing bugs of the endemic triatomine species *Mepraia spinolai*, obtained from a laboratory colony (Faculty of Science, University of Chile, Santiago, Chile). All engorged kissing bugs were kept in vials in a climate chamber (28°C, relative humidity 75%) for 40 days to enable *T. cruzi* multiplication in instances of infection. We then froze kissing bugs for 48 hours and extracted intestines and feces. After lizards were euthanized, we extracted their tissues (blood, bone, and fat) and organs (heart, stomach, intestine, lung, liver, spleen, and gonads) when possible. We stored all samples at −20°C.

We isolated whole genomic DNA from lizard and kissing bug samples by using the DNeasy Blood and Tissue Kit (QIAGEN, https://www.qiagen.com) according to manufacturer instructions. We performed real-time PCR specific for a nuclear segment of a repetitive genomic DNA sequence of *T. cruzi* DNA by using the primers Cruzi 1 and Cruzi 2 (*11*). The reaction was performed by using the Hot FIREPol EvaGreen qPCR Mix (Solis Biodyne, https://solisbiodyne.com), 0.4 µmol/L of primers, and 5 μL of template in a final volume of 20 μL. Cycling conditions were 95°C for 15 min, followed by 50 cycles at 95°C for 15 s, 65°C for 20 s, and 72°C for 20 s, which resulted in a default melting curve. We used water as a nontemplate control and DNA from a *T. cruzi* culture (Institute of Biomedical Sciences, University of Chile, Santiago, Chile) as a positive control. Each sample was analyzed in duplicate and considered positive when >1 of the replicates had specific amplification and a cycle threshold (Ct) value <40.0 (*12*).

We submitted >1 amplicons/sampled animal that had a band visualized by electrophoresis for sequencing of both strands by Macrogen (https://www.macrogen.com). We checked quality of sequences by inspection of each chromatogram, obtained the consensus sequence by using Bioedit 7.0.4.1 (*13*), and compared sequences with those available in GenBank. To assess if sequences were more similar to other trypanosomatid species, we compared sequences against a custom database that included other trypanosomatids with a full reference genome available (*T. brucei*, *T. conorhini*, *T. grayi*, *T. rangeli*, and *T. theileri*), excluding *T. cruzi*.

**Figure 1:**
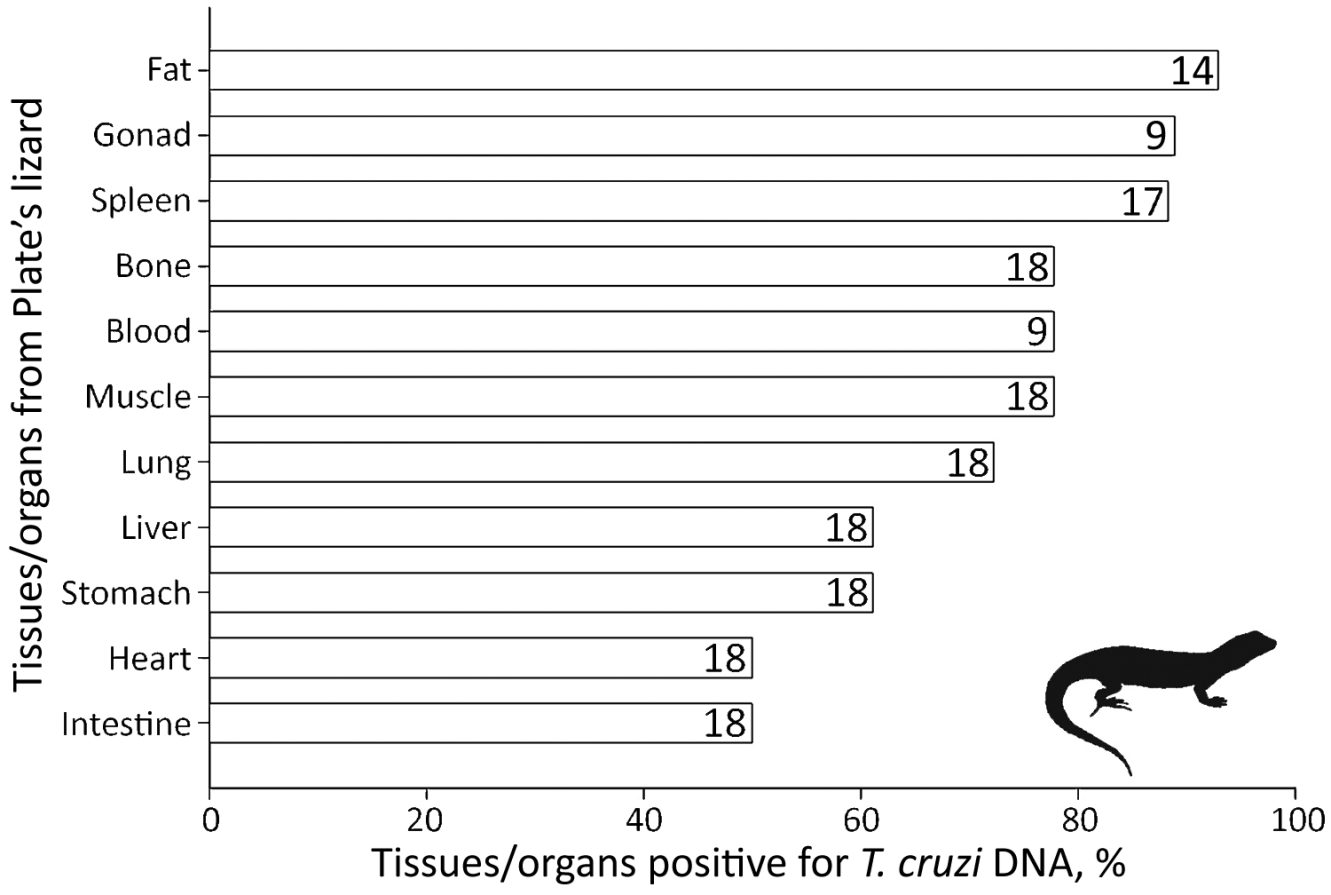
Tissues/organs tested for *Trypanosoma cruzi* infection and their percentages of infection in Plate’s lizards (*Liolaemus platei*) in study of lizards as silent hosts of *T. cruzi*. Numbers in each bar indicate number of lizards from which a specific tissue/organ was extracted and tested.

We detected *T. cruzi* infection in nearly all lizard species analyzed (Table), but not in all tested tissues or organs of *L. platei* lizards (Figure 1) and not in individual lizards of the other species (Appendix Table 1). We detected *T. cruzi* DNA in 11/13 blood samples from the Pacific Atacama racerunner (mean ± SD Ct 36.84 ± 1.47). All 18 Plate’s lizards had *T. cruzi* DNA (Ct 34.28 ± 2.57) in blood or heart when blood was not available). All 3 Dark lizards had a *T. cruzi* infection (Ct 32.42 ± 0.90) in blood or heart when blood was not available. All 10 Chilean marked geckos had *T. cruzi* DNA (Ct 32.30 ± 1.97) in heart. 

**Table:** Lizard species from southwestern South America tested for *Trypanosoma cruzi* infection, 2011–2019*

Lizard species	Common name	No. infected/no. tested	Infected tissue or organ†	Competence (range)
*Microlophus atacamensis*	Pacific Atacama racerunner	11/13	Blood	ND
*Liolaemus platei*	Plate's lizard	18/18	Liver, spleen, stomach, intestine, lung, heart, fat, muscle, bone, gonad, blood	96.43 (50–100)
*Liolaemus fuscus*	Dark lizard	3/3	Liver, spleen, stomach, intestine, lung, heart, fat, muscle, bone, gonad, blood	ND
*Garthia gaudichaudii*	Chilean marked gecko	10/10	Liver, stomach, intestine, lung, heart, muscle, bone	ND

*Competence was assessed by real-time PCR on xenodiagnostic triatomine nymphs in *Liolaemus platei* lizard only. ND, not done. †Not all types of organs were obtained for all sampled lizards (Appendix Table 1).

Results from standard sequencing showed all samples matched *T. cruzi* sequences (98.66% mean identity and 99.29% mean query cover). We detected no match between the samples and available reference genomes from other trypanosomatids. We submitted sequences to GenBank (access nos. OM730035‒75) and compiled complete BLAST analysis results (i.e., score, query cover, percentage of identity, and GenBank access number) (Appendix Table 2).

We tested Plate’s lizard competence (i.e., mean percentage of kissing bugs becoming infected after feeding on infected lizards) for 14 lizards. Nearly all (27/28) triatomine nymphs (Table) that fed on *L. platei* lizards were infected (mean ± SD Ct 33.97 ± 1.00; Appendix Table 3).

## Conclusions

**Figure 2:**
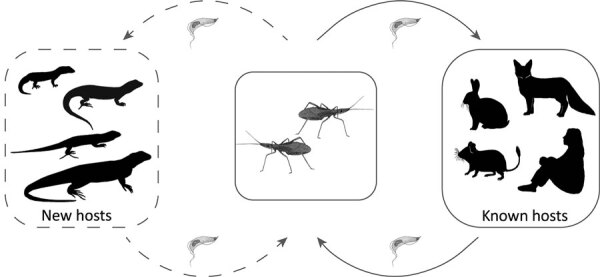
Transmission cycle of *Trypanosoma cruzi* parasites in the arid‒semiarid Mediterranean ecosystem of South America. Solid lines indicate known *T. cruzi* transmission between mammal hosts and kissing bugs, and dashed lines indicate transmission between lizards (newly described hosts) and kissing bugs.

We show that some lizard species from southern South America can be infected by *T. cruzi*; >1 species is a competent host for transmitting the protozoan to kissing bugs. This reptile group is part of the transmission cycle of Chagas disease (Figure 2), highlighting the role reptiles might have in other neglected vectorborne diseases, such as leishmaniasis and African trypanosomiasis (*7*). However, it is not clear whether lizards are infected with *T. cruzi* by kissing bug consumption, vectorborne transmission, or both.

It is crucial to assess the contribution of lizards to *T. cruzi* transmission in the sylvatic and domestic cycles of Chagas disease. Lizards might not only be competent hosts transmitting the protozoan to kissing bugs but can also be part of the diet of domestic carnivores (e.g., cats and dogs) (*14*), implying that transmission could be maintained by the presence of this new group of hosts being prey for domestic animals (*15*). Determining the threats associated with new host species and vulnerability of persons living in rural areas or in low-income countries will help evaluate transmission risk to humans and generate adequate control strategies.

Appendix Additional information on lizards as silent hosts of *Trypanosoma cruzi.*
