# Supplementary material for: Lizards as Silent Hosts of Trypanosoma cruzi
Source: Emerg Infect Dis. 2022 Jun;28(6):1250–3. doi: 10.3201/eid2806.220079 (PMC9155887; doi:10.3201/eid2806.220079)
Supplement: Appendix — Additional information on lizards as silent hosts of Trypanosoma cruzi. [file 22-0079-Techapp-s1.pdf]

Article DOI: https://doi.org/10.3201/eid2806.220079

Lizards as Silent Hosts of *Trypanosoma cruzi*

Appendix

Appendix Table 1. Cycle threshold values of real-time PCR performed in tissues/organs tested for *Trypanosoma cruzi* infection in lizard species (*Microlophus atacamensis*, *Liolaemus platei*, *L. fuscus*, and *Garthia gaudichaudii*)*

Code	Species	Blood	Heart	Stomach	Intestine	Lung	Liver	Muscle	Bone	Spleen	Fat	Gonad
M1	*M. atacamensis*	35.192†	NT	NT	NT	NT	NT	NT	NT	NT	NT	NT
M2	*M. atacamensis*	–	NT	NT	NT	NT	NT	NT	NT	NT	NT	NT
M3	*M. atacamensis*	35.406†	NT	NT	NT	NT	NT	NT	NT	NT	NT	NT
M4	*M. atacamensis*	38.868†	NT	NT	NT	NT	NT	NT	NT	NT	NT	NT
M5	*M. atacamensis*	34.719†	NT	NT	NT	NT	NT	NT	NT	NT	NT	NT
M6	*M. atacamensis*	36.797†	NT	NT	NT	NT	NT	NT	NT	NT	NT	NT
M7	*M. atacamensis*	–	NT	NT	NT	NT	NT	NT	NT	NT	NT	NT
M8	*M. atacamensis*	38.623†	NT	NT	NT	NT	NT	NT	NT	NT	NT	NT
M9	*M. atacamensis*	36.889†	NT	NT	NT	NT	NT	NT	NT	NT	NT	NT
M10	*M. atacamensis*	37.966†	NT	NT	NT	NT	NT	NT	NT	NT	NT	NT
M11	*M. atacamensis*	38.469†	NT	NT	NT	NT	NT	NT	NT	NT	NT	NT
M12	*M. atacamensis*	35.955†	NT	NT	NT	NT	NT	NT	NT	NT	NT	NT
M13	*M. atacamensis*	36.299†	NT	NT	NT	NT	NT	NT	NT	NT	NT	NT
R1	*L. platei*	34.030	–	33.192	–	–	–	35.948	–	34.686	NT	NT
R2	*L. platei*	NT	–	31.976†	–	33.653†	33.937	30.339†	33.496	33.250	28.718†	NT
R3	*L. platei*	32.920	34.256	36.766	37.355	33.028	37.943	35.111	37.468	32.876	37.228	–
R4	*L. platei*	–	32.585	35.808	35.139	26.094†	33.012	32.445†	32.844	–	35.073	32.032†
R5	*L. platei*	NT	38.915	–	–	39.497	37.643	35.828	–	38.913	35.648	38.923
R6	*L. platei*	35.863	–	35.303†	–	34.250	39.268	38.099	33.482	34.438	36.690	34.974
R7	*L. platei*	32.171	37.234	35.180	37.264†	32.794	36.594	35.838	34.718	34.017	34.897	NT
R8	*L. platei*	–	–	36.194	38.747	–	36.194	36.616	31.577†	35.471	34.747	39.461
R9	*L. platei*	30.653	30.756†	–	34.985	37.226	35.923	32.127†	32.763	31.655	32.620	30.559†
R10	*L. platei*	36.440	34.424	35.054	35.988	32.990	–	32.909	31.727†	35.231	33.073	32.507†
R11	*L. platei*	NT	33.698	37.164	–	35.187	37.457	–	36.683	36.032	36.062	NT
R12	*L. platei*	NT	38.300	–	–	36.232	37.834	–	38.486	NT	NT	NT
R13	*L. platei*	32.342	–	34.846	37.441	–	37.703	36.323	35.463	35.988	33.904	33.869
R14	*L. platei*	NT	–	–	–	37.995	–	–	–	35.431	35.607	NT
R15	*L. platei*	NT	–	–	38.563	–	–	–	–	35.946	NT	NT
R16	*L. platei*	NT	33.492	–	–	–	–	36.515	34.635	35.765	33.609	37.233
R17	*L. platei*	NT	–	36.939	37.152	37.096	–	36.288	37.286	35.560	NT	NT
R18	*L. platei*	NT	–	–	–	36.200	–	38.389	36.760	–	–	NT
R19	*L. fuscus*	NT	31.851†	32.941	32.447	34.583	31.017	33.666	32.269	32.070	33.393	NT
R20	*L. fuscus*	33.463	30.064	30.531	30.640†*	29.592	30.392	30.153	30.728	30.078	29.709	32.191
R21	*L. fuscus*	31.958	30.822	32.965	34.295	31.455†	31.775	29.609	29.129	31.426	NT	30.34
R22	*G. gaudichaudii*	NT	30.357	31.382	30.738*	32.026	33.285	34.627	33.534	NT	NT	NT
R23	*G. gaudichaudii*	NT	32.436†	32.908	34.930	33.456	33.223	33.863	30.226	NT	NT	31.867
R24	*G. gaudichaudii*	NT	29.942†	31.970	34.882	31.090	33.232	30.424	30.128	NT	NT	NT
R25	*G. gaudichaudii*	NT	34.307	30.295	33.322	31.559†	31.038	33.171	30.996	NT	NT	NT
R26	*G. gaudichaudii*	NT	33.552	33.635	33.689	32.148†	32.284	32.577	31.736	NT	NT	NT
R27	*G. gaudichaudii*	NT	31.130	34.587	31.309†	31.458	30.303	29.250	28.874	NT	NT	NT
R28	*G. gaudichaudii*	NT	29.524	28.178	30.323†	29.536	30.577	31.402	30.749	NT	NT	NT

Code	Species	Blood	Heart	Stomach	Intestine	Lung	Liver	Muscle	Bone	Spleen	Fat	Gonad
R29	*G. gaudichaudii*	NT	32.881	32.939	32.913	30.878†	34.206	31.447	34.032	NT	NT	NT
R30	*G. gaudichaudii*	NT	35.271	37.863	36.830	34.066†	–	35.291	35.055	NT	NT	NT
R31	*G. gaudichaudii*	NT	33.612†	32.676	35.089	33.657	35.333	32.727	36.036	NT	NT	NT

*Samples were considered positive when at least one of the replicates had specific amplification with a cycle threshold value <40.0. NT, nontested organs; –, absence of infection.
†Sequenced samples.

Appendix Table 2. BLAST analysis results to identify *Trypanosoma cruzi* infection in tissues and organs of 4 lizard species (*Microlophus atacamensis*, *Liolaemus platei*, *L. fuscus*, and *Garthia gaudichaudii*)

Code	Sampled species	Tissue or organ	Sequence length, bp	GenBank access number	Score	Query cover, %	Identity, %	Species
M1	*M. atacamensis*	Blood	164	OM730035	281	100	97.56	*Trypanosoma cruzi*
M3	*M. atacamensis*	Blood	130	OM730036	230	100	98.47	*Trypanosoma cruzi*
M4	*M. atacamensis*	Blood	164	OM730037	281	100	97.58	*Trypanosoma cruzi*
M5	*M. atacamensis*	Blood	164	OM730038	303	100	100.0	*Trypanosoma cruzi*
M6	*M. atacamensis*	Blood	164	OM730039	298	100	99.39	*Trypanosoma cruzi*
M8	*M. atacamensis*	Blood	164	OM730040	298	100	99.39	*Trypanosoma cruzi*
M9	*M. atacamensis*	Blood	164	OM730041	298	100	99.39	*Trypanosoma cruzi*
M10	*M. atacamensis*	Blood	164	OM730042	298	100	99.39	*Trypanosoma cruzi*
M11	*M. atacamensis*	Blood	164	OM730043	292	100	98.78	*Trypanosoma cruzi*
M12	*M. atacamensis*	Blood	164	OM730044	292	100	98.78	*Trypanosoma cruzi*
M13	*M. atacamensis*	Blood	164	OM730045	276	100	96.99	*Trypanosoma cruzi*
R2C	*L. platei*	Stomach	171	OM730046	298	95	99.33	*Trypanosoma cruzi*
R2E	*L. platei*	Lung	161	OM730047	281	100	98.14	*Trypanosoma cruzi*
R2G	*L. platei*	Fat	168	OM730048	303	99	99.40	*Trypanosoma cruzi*
R2H	*L. platei*	Muscle	168	OM730049	302	98	99.40	*Trypanosoma cruzi*
R4E	*L. platei*	Lung	166	OM730050	307	100	100.0	*Trypanosoma cruzi*
R4H	*L. platei*	Muscle	166	OM730051	296	98	99.39	*Trypanosoma cruzi*
R4J	*L. platei*	Gonads	168	OM730052	307	98	100.0	*Trypanosoma cruzi*
R6C	*L. platei*	Stomach	166	OM730053	307	100	100.0	*Trypanosoma cruzi*
R7D	*L. platei*	Intestine	167	OM730054	307	99	100.0	*Trypanosoma cruzi*
R8I	*L. platei*	Bone	166	OM730055	279	98	97.55	*Trypanosoma cruzi*
R9B	*L. platei*	Spleen	167	OM730056	292	100	98.20	*Trypanosoma cruzi*
R9F	*L. platei*	Heart	166	OM730057	291	98	98.77	*Trypanosoma cruzi*
R9H	*L. platei*	Muscle	167	OM730058	276	98	96.95	*Trypanosoma cruzi*
R9J	*L. platei*	Gonad	169	OM730059	294	97	98.79	*Trypanosoma cruzi*
R9S	*L. platei*	Blood	166	OM730060	298	98	99.39	*Trypanosoma cruzi*
R10I	*L. platei*	Bone	167	OM730061	307	99	100.0	*Trypanosoma cruzi*
R10J	*L. platei*	Gonad	166	OM730062	307	100	100.0	*Trypanosoma cruzi*
R19F	*L. fuscus*	Heart	164	OM730063	287	100	98.17	*Trypanosoma cruzi*
R20D	*L. fuscus*	Intestine	164	OM730064	303	100	100.0	*Trypanosoma cruzi*
R21E	*L. fuscus*	Lung	164	OM730065	287	100	98.17	*Trypanosoma cruzi*
R22D	*G. gaudichaudii*	Intestine	164	OM730066	281	100	97.56	*Trypanosoma cruzi*
R23F	*G. gaudichaudii*	Heart	164	OM730067	303	100	100.0	*Trypanosoma cruzi*
R24F	*G. gaudichaudii*	Heart	164	OM730068	270	100	96.34	*Trypanosoma cruzi*
R25E	*G. gaudichaudii*	Lung	164	OM730069	289	100	97.77	*Trypanosoma cruzi*
R26E	*G. gaudichaudii*	Lung	164	OM730070	298	100	99.39	*Trypanosoma cruzi*
R27D	*G. gaudichaudii*	Intestine	164	OM730071	270	98	96.89	*Trypanosoma cruzi*
R28D	*G. gaudichaudii*	Intestine	164	OM730072	298	100	99.39	*Trypanosoma cruzi*
R29E	*G. gaudichaudii*	Lung	164	OM730073	287	100	98.17	*Trypanosoma cruzi*
R30E	*G. gaudichaudii*	Lung	164	OM730074	250	98	94.44	*Trypanosoma cruzi*
R31F	*G. gaudichaudii*	Heart	164	OM730075	281	100	97.56	*Trypanosoma cruzi*

Appendix Table 3. Cycle threshold values of real-time PCR performed in second-stage *Mepraia spinolai* nymphs used in xenodiagnosis assays to test for *Trypanosoma cruzi* infection in Plate’s lizard (*Liolaemus platei*)*

	Xenodiagnostic triatomines			
Lizard code	Nymph 1	Nymph 2	Nymph 3	Competence, %
R1	34.939	33.791	34.770	100
R2	34.499	34.140	33.825	100
R3	32.569	35.288	31.803	100
R4	34.834	NF	NF	100
R5	33.756	33.110	NF	100
R6	34.051	34.922	NF	100
R9	35.792	NF	NF	100
R10	35.307	NF	NF	100
R11	35.015	33.829	NF	100
R14	33.705	33.613	NF	100
R15	32.526	NF	NF	100
R16	–	33.935	NF	50
R17	33.696	31.912	NF	100
R18	32.454	32.810	32.416	100

*NF, nymph not feeding on the lizard specimen or eaten by the lizard; –, absence of infection. Competence is shown as percentage of nymphs that became infected after feeding on an infected lizard specimen.
